# Development of two multiplex PCR assays for rapid detection of eleven Gram-negative bacteria in children with septicemia

**DOI:** 10.1186/s41182-024-00606-3

**Published:** 2024-06-05

**Authors:** Gabriel Miringu, Abednego Musyoki, Betty Muriithi, Ernest Wandera, Dan Waithiru, Erick Odoyo, Hisashi Shoji, Nelson Menza, Yoshio Ichinose

**Affiliations:** 1 Kenya Medical Research Institute, Institute of Tropical Medicine, Nagasaki University, Nairobi, 19993-00202 Kenya; 2 Department of Medical Laboratory Sciences, Kenyatta University, Nairobi, Kenya; 3 Center for Virus Research, KEMRI, Nairobi, Kenya; 4 Center for Microbiology Research, KEMRI, Nairobi, Kenya; 5 United States Army Medical Research Unit, KEMRI, Nairobi, Kenya; 6 Embassy of Japan, La Paz, Bolivia

**Keywords:** Septicemia, mPCR, Gram-negative bacteria, Diagnosis

## Abstract

**Aim:**

This study aimed to develop a multiplex PCR assay for simultaneous detection of major Gram-negative etiologies of septicemia and evaluate its performance.

**Methods:**

Multiplex PCR (mPCR) assays were developed targeting 11 bacterial strains. Species-specific primers were confirmed using known clinical isolates and standard strains. Gradient PCR was performed on each primer against its target bacterial gene to determine its optimal amplification condition. The minimum detectable DNA concentration of the two assays was evaluated by adjusting bacterial DNA concentration to 100 ng/μL and, tenfold serially diluting it up to 10 pg/μL with DNAse-free water. The diagnostic accuracy of mPCR assays was established by subjecting the assays to 60 clinical blood samples.

**Results:**

Two mPCR assays were developed. Optimal primer annealing temperature of 55 °C was established and utilized in the final amplification conditions. The assays detected all targeted bacteria, with a 100 pg minimum detectable DNA concentration. Pathogens were not detected directly from whole blood, but after 4 h and 8 h of incubation, 41% (5/12) and 100% (12/12) of the bacteria were detected in culture fluids, respectively. The assays also identified *Salmonella* spp*.* and *Klebsiella pneumoniae* co-infections and extra pathogens (1 *E. coli* and 2 *K. pneumoniae*) compared with culture. The sensitivity and specificity of the mPCR were 100.0% (71.7–100.0) and 98.0% (90.7–99.0), respectively. The area under the ROC curve was 1.00 (1.00–1.00).

**Conclusions:**

The mPCR assays demonstrated substantial potential as a rapid tool for septicemia diagnosis alongside the traditional blood culture method. Notably, it was able to identify additional isolates, detect co-infections, and efficiently detect low bacterial DNA loads with high sensitivity, implying its value in enhancing efficiency of diagnosis of septicemia.

## Introduction

Septicemia is an invasion of the bloodstream by bacteria originating from other body organs that remains a threat to public health worldwide [1]. Although the burden of septicemia is not well-elucidated, prevalence in most low-income settings ranges between 2 and 15% [2–5]. The vast majority of morbidity and mortality due to septicemia occur in low-and-middle-income countries [4, 6], with malnutrition and poor health infrastructure increasing susceptibility [7]. Children below 5 years and the elderly are disproportionately affected [8]. Even though advancements in treatment and critical care have substantially improved the management of septicemia, morbidity is still high in most high-burden countries, and global under-five mortality targets due to septicemia are not yet achieved [6]. Inadequate sensitivity of available diagnostic approaches makes early detection extremely difficult and largely accounts for undesirable outcomes. Delays in initiation of treatment increases the likelihood of mortality and may have serious consequences [9], especially in neonates [10]. A rapid and sensitive diagnostic test is, therefore, a priority, not only to improve patient outcomes but also to achieve antimicrobial stewardship.

Etiologies of septicemia include both Gram-positive bacteria (GPB) and Gram-negative bacteria (GNB) [11, 12], epidemiologically differing by region, gender and age [13, 14]. GNB, including *Klebsiella pneumoniae*, *Escherichia coli*, *Proteus* spp., *Pseudomonas* spp., *Enterobacter* spp., *Citrobacter* spp., and *Acinetobacter* spp., have been identified as the most frequent causes of septicemia, particularly among neonates and younger children [15, 16]. Most importantly, they are increasingly associated with septic shock [15, 17], with an elevated likelihood of developing antibiotic resistance [19], and generally poor clinical outcomes [20]. Therefore, timely and accurate diagnosis is critical for improved patient management.

Diagnosis of septicemia mainly depends on detecting causative microorganisms in a blood sample through blood culture, which usually takes up to 5 days for potential causative organisms to grow, be identified and profile their sensitivity to antibiotics. Given the importance of early and adequate initiation of antibiotic therapy for patients with septicemia, blood culture falls short of timeliness, a crucial element of an ideal diagnostic test. Moreover, its accuracy is affected by fastidious microorganisms, contaminants or prior exposure to antibiotics [11]. Furthermore, inadequate blood sample volume coupled with the transient or intermittent nature of septicemia can complicate diagnosis in children [12].

Several novel technologies for septicemia diagnosis, based on molecular techniques of detecting pathogens at strain level, have been developed to circumvent blood culture shortfalls [13]. Although these tools have high initial and operation costs, their potential to address gaps in pathogen identification and susceptibility to antibiotics makes them a viable alternative to conventional methods. Initial technologies were predominantly singleplex PCR-based tools, whose main shortfall was their inability to identify co-infections, given the polymicrobial nature of septicemia [14]. Consequently, several multiplex PCR assays have been developed [15], with the advantage of broad-range microbial detection, that are more suitable for the clinical setting. However, the broad-range target compromises their sensitivity [16, 17], and the panels may not contain all clinically relevant pathogens.

This study developed two sets of mPCR assays to detect 11 (Table 1) select GNB etiologies of septicemia in children. The 11 organisms were selected based on unpublished data from ongoing surveillance within the study area, and other studies in Kenya [18, 19] and in the region [20, 21] that have also frequently observed these organisms among children with septicemia.

**Table 1:** Target and off-target strains

Strain name	Strain identifier
Target reference strain	
Multiplex A	
*Acinetobacter baumannii* K001-AC	ATCC 19606
*Escherichia coli*	ATCC 25922
*Pseudomaonas aeruginosa*	ATCC 27853
*Salmonella typhi*	ATCC 19430
*Shigella group*	ATCC 23354
*Vibrio cholerae toxR (K14-1980)*	ATCC 11623
Multiplex B	
*Aeromonas hydrophila*	ATCC 35654
*Campylobacter jejuni*	ATCC 49943
*Enterobacter cloacae* complex BE.108	ATCC 35030
*Klebsiella pneumoniae* KP-7	ATCC 13883
*Providentia alcalifaciens*	ATCC 9886
Off-target reference strain	
*Citrobacter freundii*	Clinical isolate
*Enterococcus faecalis*	Clinical isolate
*Proteus mirabillis*	Clinical isolate
*Phingomonas paucimobilis*	Clinical isolate
*Streptococcus pneumoniae*	Clinical isolate

## Materials and methods

### Bacterial strains

Eleven target standard reference strains purchased from the American Type Culture Collection [American type culture collection (ATCC)] (Manassas, VA, USA) and five off-target strains (Table 1) were selected for confirmation of species primers. The off-target strains were well-characterized clinical isolates from Kiambu and Homabay Counties in Kenya that were stored at − 80 °C in the NUITM–KEMRI Project, Kenya Research Station. Target bacterial stocked strains (Table 2) for confirming species-specific primers were also identified.

**Table 2:** Stocked laboratory confirmed clinical isolates

Isolate	No. of isolates	Isolate	Number of isolates
*K. pneumoniae*	10	*Salmonella typhimurium*	3
*P. aeruginosa*	10	*Salmonella paratyphi B*	2
*Enterobacter cloacae*	6	*Campylobacter jejuni*	4
*E. coli*	20	*Aeromonas sobria*	3
*Salmonella typhi*	2	*Acinetobacter baumannii*	4
*Salmonella enteritidis*	6	*Aeromonas hydrophilia*	5
*V. cholerae*	12	*Shigella sonnei*	3
*Providentia alcalifaciens*	5	*Shigella dysenteri*	2
*Campylobacter coli*	3	*Shigella flexineri*	1

To revive the strains, the stocked isolates were transferred from − 80 °C into ice. A loopful of glycerol stock was streaked on LB-Agar medium, and incubated at 37 °C for 18–24 h. The identity of all isolates using the automated identification system VITEK-2 GN cards (Biomeriux, France) and monoplex PCR.

### Primer selection and design

We used ten sets of primers (Table 3), of which six were previously published sets targeting known species-specific genes. Remaining four sets were designed for this study based on the conserved regions, using online primer design software (https://bioinfo.ut.ee/primer3/). The study utilized primers with no analytical cross-reactivity, according to in silico multiple sequence alignment in MPprimer software [22], and synthesized by Sigma (Sigma-Aldrich, Germany).

**Table 3:** Selected and designed primers with expected amplicon sizes

Pathogen	Target gene	Primer sequence (5′–3′)	Amplicon size (bp)	Source
Multipex assay A				
*Salmonella* spp.	*InvA-F*	GTGAAATTATCGCCACGTTCGGGCAA	284	[37]
	*InvA*-R	TCATCGCACCGTCAAAGGAACC		
*Escherichia coli*	*uspA*-F	TTCACATCAATAAGCCCGGT	128	This study
	*uspA*-R	CTCTCCCCGGAAAGCAAAGT		
*Shigella group*	*uspA*-F	TTCACATCAATAAGCCCGGT	128	This study
	*uspA*-R	CTCTCCCCGGAAAGCAAAGT		
*Acinetobacter baumanii*	*secE-*F	GACGCGCCAATTCCTCAAAG	320	This study
	secE-R	TGCCACGATGTTGTGACTGT		
*Pseudomonas aeruginosa*	*gyrB-F*	GGCGTGGGTGTGGAAGTC	190	[38]
	gyrB-R	TGGTGGCGATCTTGAACTTCTT		
*Vibrio cholerae*	toxR-F	ATGTTCGGATTAGGACAC	883	[39]
	toxR-R	TACTCACACACTTTGATGGC		
Multiplex assay B				
*Camphylobacter* spp.	flaA-F	AATAAAAATGCTGATAAAACAGGTG	855	[40]
	flaA-R	TACCGAACCAATGTCTGCTCTGATT		
*Enterobacter* spp.	ampCgene-F	TTGACTCGCTATTACGGAAGAT	1181	[41]
	ampCgene-R	GCAATGTTTTACTGTAGCGC		
*Aeromonas* spp.	DnaJ gene Aero -F	CGAGATCAAGAAGGCGTACAAG	891	[42]
	dnaJ gene Aero -R	CACCACCTTGCACATCAGATC		
*Providentia alcalifacience*	16S rRNA-F	ACCGCATAATCTCTTAGG	515	This study
	16S rRNA-R	CTACACATGGAATTCTAC		
*Klebsiella pneumonia*	gltA-F	CGTCGTGCGAAAGACAAGA	174	This study
	gltA-R	GCGATATGCTCCAGCTCCAT		
Confirmation primer for *E. coli*				
*E. coli*	EClpma (− 1)	ACCAGACCCAGCACCAGATAAG	463	[23]
	EClpma (+ 1)	GCACCTACGATGTTTTTGACCA		

### Bacterial DNA extraction

Bacterial DNA from clinical stocked and standard strains in 250 µL broth Luria–Bertani (LB) were extracted using the QIAamp DNA Kit (Qiagen, Netherlands), following the manufacturer’s instructions. The resultant DNA quantity and purity were determined using Qubit and NanoDrop 2000, (Thermo Scientific, USA) spectrophotometers.

### Development of mPCR assays

Initially, this study determined the primer’s annealing temperature by running monoplex PCR assays for each primer pair using the gradient PCR approach, which were ranging between 52 and 58 °C. Assays were of 25 μL reaction volume, comprising of Illustra™ Ready-To-Go™ Beads (GE Healthcare-life science), molecular grade water (22 μL), DNA template (2 μL), and specific primer (1 μL) of 10 pmol/μL working concentration solution. PCR amplifications were performed using Biorad-iCycleriQ thermocycler (Bio-Rad, USA) with the following conditions: initial denaturation at 95 °C for 3 min; 35 cycles of denaturation at 95 °C for 30 s, annealing at 52–58 °C for 45 s and extension at 72 °C for 1 min, and final extension at 72 °C for 7 min. PCR products were observed by minigel electrophoresis using 2% agarose gel with Tris Borate EDTA (TBE) buffer at 100 V for 30 min.

For optimizing the mPCR assays, two specific primer pairs were mixed, amplified and their product sizes evaluated. A primer set was added consecutively until all other primer sets were mixed, and optimal amplification confirmed. Amplification conditions were the same as those of monoplex assays, but the concentration of a primer pair used at each stage was adjusted based on the previous primer pair product. To minimize overcrowding of PCR product bands and to increase the accuracy of the mPCR, the specific targets were grouped into two sets as follows: *Salmonella* spp., *Escherichia coli*, *Shigella* spp., *Acinetobacter baumannii*, *Pseudomonas aeruginosa*, and *Vibrio cholerae* in mPCR A assay and *Campylobacter* spp., *Enterobacter* spp., *Aeromonas* spp., *Providentia alcalifaciens*, and *Klebsiella pneumoniae* in mPCR B assay. Results of these the two mPCR assays were confirmed by running singleplex PCRs using single primer sets.

### Analytical performance of the two mPCR assays

To evaluate the limit of detection (LOD) of the mPCR assays, the concentration of DNA template for each target reference strain was adjusted to 100 ng/μL and tenfolds serially diluted up to 10 pg/μL using DNAse-free water. The serial dilution of each target DNA template was then amplified by the optimized mPCR reaction system. To evaluate the reproducibility of the assays, each serial dilution was prepared in triplicates. mPCR inter-assay was performed on each dilution.

### Performance of the multiplex assays on clinical samples

Performance of the mPCR assays was evaluated by subjecting them to clinical venous blood samples collected from 60 children aged below 5 years. These were children presenting with septicemia symptomatically (at least two of the following signs or symptoms, including fever (temp > 38 °C or < 36 °C), WBC > 12,000 cells/mm^3^, or < 4000 cells/mm^3^, or bands > 10%, respiratory Rate > 24 breaths/min, and Heart Rate > 90 beats/min) at Kiambu County Referral Hospital, Kenya. Approximately 8 mL of venous blood was collected from each participant. One ml aliquot of blood was put in an EDTA tube and stored at 4 °C for bacterial DNA extraction for mPCR assays, and 3 mL of blood put in duplicates in a biphasic blood culture medium (Himedia, India) and immediately transported to the NUITM laboratory for analysis.

The biphasic blood cultures were incubated aerobically at 37 °C and microaerophilically at 42 °C for up to 5 days, with subculture after every 24 h on MacConkey’s agar (Himedia, India), Xylose Lysine Deoxycholate (XLD) agar (Himedia, India), Thiosulfate-Citrate-Bile Salts-Sucrose (TCBS) agar (Eiken Chemicals, Japan) for *V. cholerae*, and Blood agar base (Oxoid, UK) with campylobacter selective supplement (Oxoid, UK) and 10% defibrinated sheep blood for *Campylobacter* spp. The subcultures were incubated for 24 h aerobically at 37 °C, except for blood agar for *Campylobacter* spp., which was done micro-aerophilically at 42 °C for the same duration. Resultant colonies were identified by Gram staining and Vitek 2 GN automatic cards identification system (Biomérieux, France).

About 0.5 mL sample culture broth was collected into 1 mL cryo-vial after every 4 h of incubation up to 20 h for DNA extraction to determine minimum detection time; the earliest timepoint the newly developed mPCR would detect target bacterial DNA in a blood culture before recovery of the isolate in subculture media. Bacterial DNA was extracted from culture broth and whole blood samples using the QIAamp DNA Blood Mini Kit (Qiagen, Netherlands), with 200 µL of the two sample types separately aliquoted into 2.0 mL sample tubes containing proteinase K on an ice block and following the manufacturer’s instructions. Before PCR amplification, Qubit™ fluorometer (Thermo Fisher Scientific™, USA) and NanoDropTm 2000 spectrophotometers were used for quantification and purity check of the harvested DNA, respectively.

### Statistical analysis

STATA Version 14 (StataCorp LLC, TX, USA) was used for statistical analysis of data. Blood culture method was used as gold standard and compared with the developed mPCRs to assess its diagnostic performance. Sensitivity, specificity, overall agreement and area under the receiver operating characteristics (ROC) curve were calculated to establish the accuracy of the mPCR assays.

## Results

### Confirmation of primer specificity

Upon subjecting selected primers to the standards and known clinical strains, all 11 monoplex PCR assays could accurately detected the target pathogens, with 100% detection rate (Table 4).

**Table 4:** Sensitivity detection rate of selected primer pairs

	Strain (clinical samples)	Number tested	number detected	Detection rate (%)
1	Pathogenic* E. coli*	16	16	100
	Non-pathogenic* E. coli*	4	4	100
2	*Shigella group*	6	6	100
3	*Salmonella* spp.	13	13	100
4	*Vibrio cholerae*	12	12	100
5	*Pseudomonas aeruginosa*	10	10	100
6	*Klebsiella pneumoniae*	10	10	100
7	*Aeromonas* spp.	8	8	100
8	*Acinetobacter baumanii*	4	4	100
9	*Enterobacter* spp.	6	6	100
10	*Providentia. alcalifaciens*	5	5	100
11	*Camphylobacter* spp.	7	7	100

### Development and optimization of the mPCR assays

**Fig. 1:**
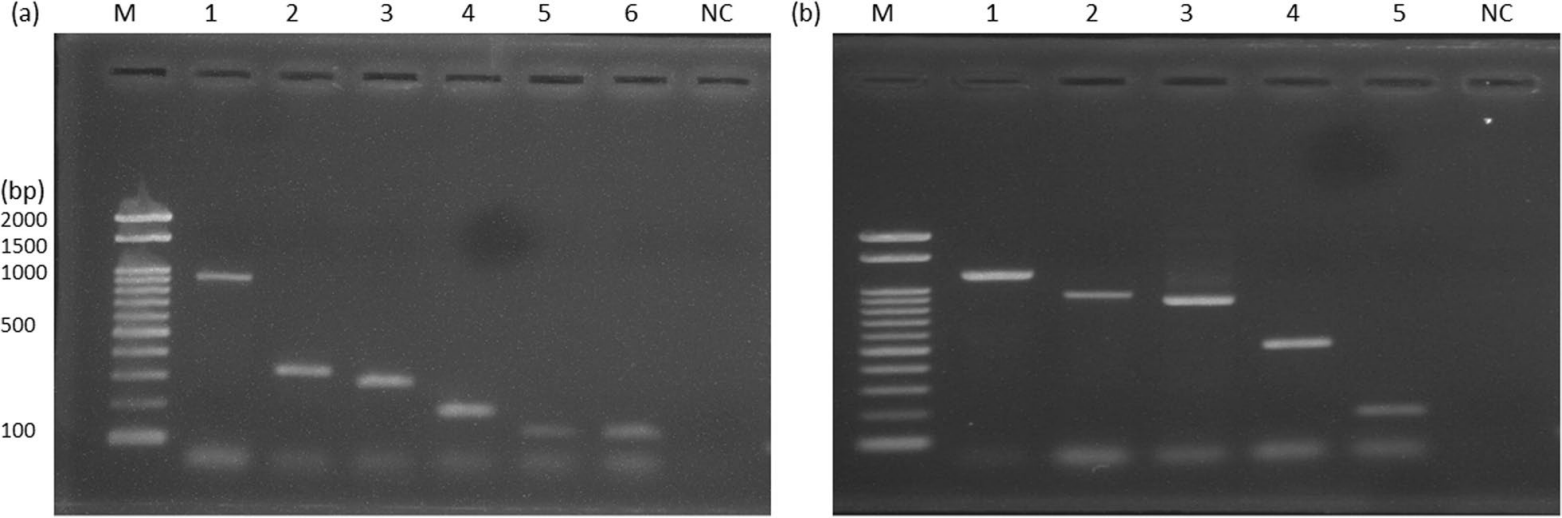
Gels showing target genes amplified at the optimized annealing temperature (55 °C). **a** Multipex A: M = Marker, Lane 1 (*Vibrio cholerae*, (883 bP), 2. *Acinetobacter baumanii* (320 bp), 3. *Salmonella* spp. (284 bp), 4. *Pseudomonas aeruginosa* (190 bp), 5. *Shigella* spp. (128 bp), 6. *E.coli*(128 bp), 7. Negative control. **b** Multiplex B: M = Marker, 1. *Enterobacter* spp. (1181 bp), 2. *Aeromonas* spp. (891 bp), 3. *Camphylobacter* spp. (855 bp), 4. *Providentia alcalifacience* (515 bp), 5*. K. pneumoniae* (174 bp), 6. Negative control

**Fig. 2:**
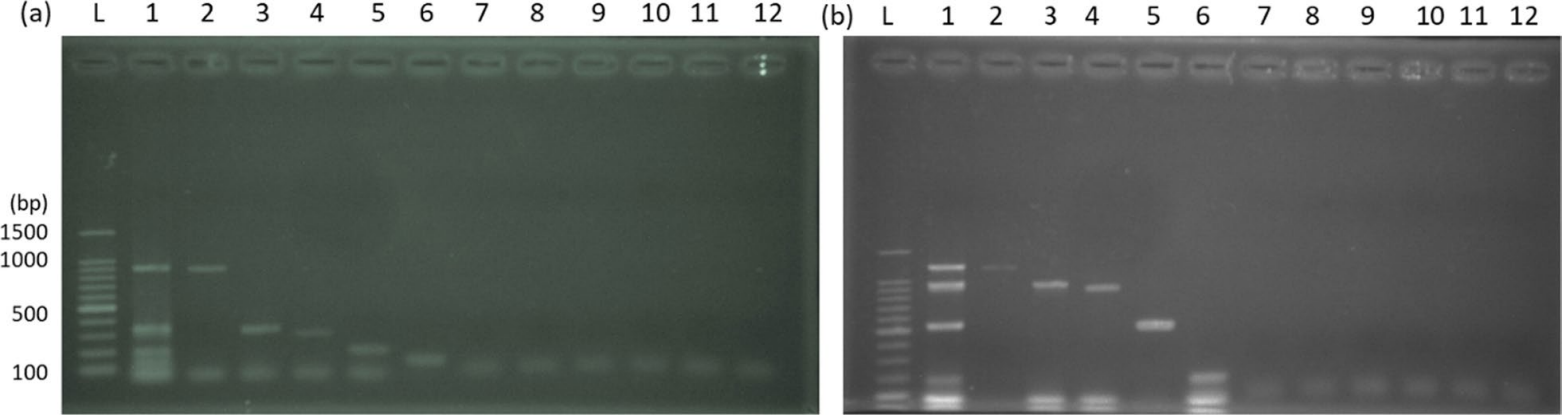
Gels showing specificity of developed Mpcr. **a** Multipex A: l = Ladder, Lane 1. DNA mix, Lane 2 (*Vibrio cholerae*, 883 bP), 3. *Acinetobacter baumanii* (320 bp), 4. *Salmonella* spp. (284 bp), 5. *Pseudomonas aeruginosa* (190 bp), 6. *E. coli* (128 bp), 7–11. Off targets (Table 1) 12. Negative control. **b** Multiplex B: L = Ladder, Lane 1 Mixed DNA, 2. *Enterobacter* spp. (1181 bp), 3. *Aeromonas* spp. (891 bp), 4. *Camphylobacter* spp. (855 bp), 5. *Providentia alcalifacience* (515 bp), 6. *K. pneumoniae* (174 bp), 7–11. Off targets (Table 1) 12. Negative control

Optimal primer annealing temperature was achieved at 55 °C using gradient PCR. Other amplification conditions were: initial denaturation at 95 °C for 3 min; 35 cycles of denaturation at 95 °C for 30 s, annealing at 55 °C for 45 s, extension at 72 °C for 1 min, and final extension at 72 °C for 7 min. The two sets of mPCRs assays simultaneously amplified all the six and five target bacterial pathogens, respectively, using the reference strains. It also produced distinct PCR products using combined bacterial lysates, except for *E. coli* and *Shigella* spp., which shared the same target gene (uspA), Fig. 1. These results demonstrated the capability of the mPCR systems to detect all 11 targeted species, whether present in their pure forms or within mixed bacterial compositions extracted from clinical specimens. These two newly developed mPCRs, accurately amplified all 11 bacterial targeted genes in a DNA mix as well as single bacterial DNA target. This assay did not show any amplification to the five off-target bacteria (Table 1, Fig. 2).

### Reproducibility and LOD of the two mPCR assays for bacterial identification

**Fig. 3:**
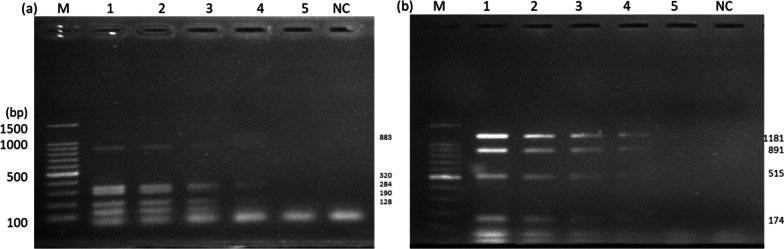
Gels showing the minimum detectable mixed bacterial DNA concentration. **a** Multiplex A mixed bacterial DNA and **b** multiplex B mixed bacterial DNA. **a**, **b** M (marker) lane 1. (100 ng), lane 2. (10 ng), lane 3. (1 ng), (lane 4. 100 pg), lane 5. (10 pg); lane 6. NC

The minimum detectable DNA concentration by the two mPCRs was 100 pg. The results of the reproducibility assessment yielded consistent outcomes. In each replicate run, concordant results for the presence or absence of the target bacterial strains were observed. No variations or discrepancies were detected among the replicates, suggesting good reproducibility of results fromthe mPCR assays, Fig. 3.

### Diagnostic performance of the two mPCR assays using blood samples compared to conventional blood culture

**Fig. 4:**
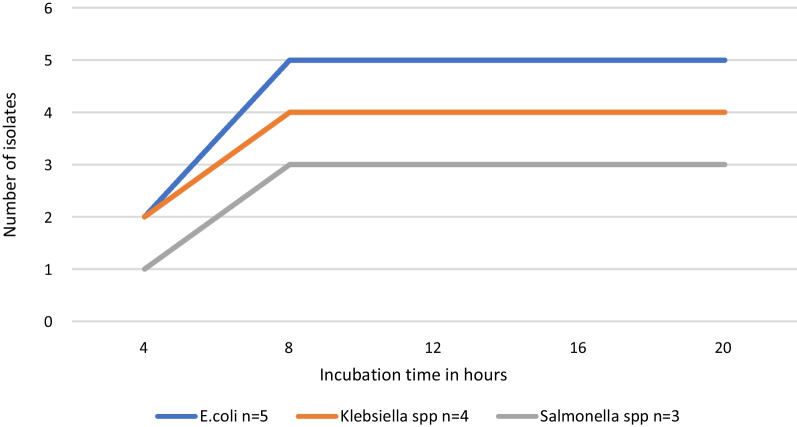
Bacterial pathogens detected in culture fluids at different incubation period

Of the 11 targeted bacterial strains, three strains were detected in patients’ blood samples by primary blood culture and mPCR, as shown in Table 5. Based on conventional culture, 15% (9/60) of the blood samples were culture-positive for mono-target bacteria, whereas 17% (10/60) were mPCR-positive. Of the ten mPCR-positive samples, two had a *Salmonella* spp., and *Klebsiella pneumoniae* co-infection. The extra detected pathogens by mPCR were 1 *E. coli* and 2 *K. pneumonia*, Table 5. The sensitivity and specificity of the mPCR using primary culture as the gold standard were 100.0% (71.7–100.0) and 98.0% (90.7–99.0), respectively (Table 5). The area under the ROC curve was 1.00 (1.00–1.00). The developed mPCR assays detected 5 pathogens (41%) out of 12 bacterial pathogens after 4 h of incubation and 12 pathogens (100%) out of 12 bacterial pathogens after 8 h of incubation (Fig. 4).

**Table 5:** Pathogens identified by primary culture and multiplex PCR

Organism	mPCR *n* = 10	Blood culture *n* = 9
*Escherichia coli*	5*	4
*Salmonella* spp.	3	3
*Klebsiella pneumoniae*	2**	2
mPCR sensitivity % (CI)	100.0 (71.7–100.0)	
mPCR specificity % (CI)	98.0 (90.7–99.9))	
Positive predictive value % (CI)	90.0 (71.4–100.0)	
Negative predictive value % (CI)	98.0 (92.9–100.0)	

Extra * one and ** two pathogens respectively detected by mPCR assays

This finding highlight capacity of the mPCR assays to bridge the gap between the advantage and limitations of blood culture, to improve diagnosis of septicemia.

## Discussion

This study developed two mPCR assays to detect 11 GNB pathogens commonly associated with septicemia in children. Nine identified at the genus level and two at the species level. *E. coli* and *Shigella* spp*.* were detected using a universal primer (uspA gene), to enhance the assays’ specificity of the assay by eliminating potential outlier annealing temperatures associated with specific *E. coli* and *Shigella* spp. genes. An identification step targeting the *lacY* gene present in *E. coli* but not in *Shigellae* [23] added to differentiate the organisms.

The assays’ sensitivity and specificity were 100% and 98%, respectively, with an area under ROC curve of 1.00. These assays were able to detect a co-infection that was missed by blood culture. Sensitivity was optimal due to superiority of the PCR technology, and its ability to differentiate organisms at genetic level, unlike the blood culture approach which relies on physiological characteristics, that may not necessarily be specific. The high sensitivity of the assays was further confirmed by the resulting area under ROC curve that denotes optimal accuracy and high capacity to discriminate between those with and without the targeted pathogen. The slightly sub-optimal specificity of the assays could have been due to susceptibility of the PCR method to contamination and false positives. Observed sensitivity and specificity were, however, consistent with previous studies which have found sensitivity of multiplex PCR tests for bacterial etiologies of blood stream infections among children to be higher than specificity [24]. Moreover, Cox et al. [25] observed equally high positivity and negativity upon subjecting blood culture fluid to a mPCR panel.

The high sensitivity and specificity of these assays are particularly noteworthy as it enhances its ability to detect target pathogens, enabling a definitive diagnosis. Furthermore, its capacity to detect multiple etiologies simultaneously increases its viability for application in clinical settings. Several septicemia molecular diagnostic tools, including SeptiFast, SepsiTest, SeptiCyte, U-dHRM, Prove-it, and Iridica Plex ID, have been developed to circumvent the accuracy and timeliness shortcomings of BC [26]. These kits, however, bear high purchase and operation costs, making their routine use in resource-limited countries, such as Kenya, almost impractical. Moreover, they rely on PCR-amplification and sequencing of microbial conserved genomic regions—ribosomal RNA genes and the 16S–23S inter-spacer regions; hence, turnaround time still remains high although better than that of BC [27]. Furthermore, the available multiplex PCR (mPCR) tools target one or two bacteria, which might be cumbersome, expensive and may underestimate the BSI aetiology [28].

Additional strength of the mPCR assays was its capability to detect DNA concentrations as low as 100 pg which was similar to the previous reports [29, 30], although these targeted different pathogens. This outcome is attributable to the methodology of PCR that enables it to amplify small concentrations to readily detectable quantities. The high limit of detection suggests capacity of the assays to achieve early and timely diagnosis of septicemia and their suitability as an adjunct to blood culture, especially in detection of bacterial pathogens that take long to reach detectable levels in culture.

The mPCR could not detect the target pathogens directly from whole blood samples, possibly due to low bacterial load, the small sample volume (250 µL), or potential inhibition by human DNA [31, 32]. An incubation step was, therefore, introduced before the bacterial DNA extraction to increase bacterial DNA yield. Pathogens were subsequently detectable after at least 4 h and at most 8 h of incubation. While this approach increased accuracy of the assays, it extended the overall testing process. Despite the extended process, the time-to-detection was still shorter than that of blood culture, suggesting the ability of the assays to increase utility of blood cultures to improve diagnosis especially in normally sterile body fluids.

On application of the assay to clinical testing, an overall agreement of 98.3% between blood culture and mPCR was observed. Agreement was higher than that observed in previous studies (60 to 80%) [33, 34]. The discordant case was a blood culture-negative and PCR-positive result. Since the mPCR did not miss organisms identified by blood culture, it is plausible that the mPCR assays were superior to blood culture. However, since the primary advantage of PCR-based assays is timeliness, blood culture still merits as the gold standard. Nevertheless, further larger studies can be conducted to ascertain clinical usefulness of the assays.

Only three species of pathogens, namely *E. coli*, *Salmonella* spp*.,* and *K. pneumoniae,* were detected from clinical samples. This finding agrees well with previous studies that have identified these bacteria as the most common etiologies of septicemia in developing countries [35, 36]. Additionally, the mPCR assays detected co-infection in two cases involving *Salmonella* spp. and *Klebsiella pneumoniae.* Therefore, the finding of this study supports the observation that mPCR can increase diagnostic yield, by detecting co-infections that would be missed by blood culture [34].

## Conclusions

The two mPCR assays demonstrated significant potential as a rapid tool for septicemia diagnosis alongside traditional blood culture method. Notably, it was able to identify additional isolates, detect co-infections, and efficiently detect low bacterial DNA loads. Validating these assays in a large and diverse study population, optimizing them to use blood samples, and incorporating GPB could improve the quality of septicemia diagnosis and patients’ management.

## Study limitation

The mPCR assays could not detect pathogens directly in whole blood, which could have substantially reduced diagnosis turnaround time, and this study did not include Gram-positive bacteria that cause septicemia.

## Data Availability

The datasets used and/or analyzed during the current study are available from the corresponding author on reasonable request.
